# Influenza vaccination in patients with juvenile idiopathic arthritis under different treatments: safety and immune response

**DOI:** 10.1186/s12969-025-01099-y

**Published:** 2025-04-25

**Authors:** Adam Gyori, Arnold Nagy, Gabor Ottoffy, Tamas Decsi, Diana Simon, Timea Berki, Timea Dergez, David Kuti, Bernadett Mosdosi

**Affiliations:** 1 Department of Pediatrics, Medical School, University of Pecs, Pecs, Hungary; 2 Department of Immunology and Biotechnology, Medical School, University of Pecs, Pecs, Hungary; 3 Institute of Bioanalysis, Medical School, University of Pecs, Pecs, Hungary; 4 Department of Virology, National Center for Public Health and Pharmacy, Budapest, Hungary

**Keywords:** Influenza vaccine, Juvenile idiopathic arthritis (JIA), Safety, Flowcytometry, Immunogenicity

## Abstract

**Background:**

Annual flu vaccination is recommended for children with rheumatic diseases. We investigated the cellular and humoral immune response and safety in pediatric patients that received inactivated influenza vaccines.

**Methods:**

This is a comparative study of in 41 children with juvenile idiopathic arthritis (JIA) receiving influenza vaccination while being treated with methotrexate (MTX) or biological therapy. The influenza vaccination was administered as a single dose of trivalent influenza vaccine (TIV). Serological tests to monitor seroconversion and seroprotection were performed at baseline and at 4 as well as 12 weeks after vaccination.

**Results:**

In all of the 41 children with JIA and the 22 healthy children seroconversion and seroprotection were observed for Influenza A. For Influenza B, no adequate seroconversion rates were not detected in any of the groups studied. No significant differences were observed in lymphocyte subpopulations when analysing time points and groups simultaneously. There were no relapses or cases of influenza infection after the vaccination. Our findings do not suggest non-specific immune activation following vaccination based on the distribution and quantity of the lymphocyte subsets that were investigated.

**Conclusion:**

The present study demonstrates adequate seroprotection rates against influenza A in immunosuppressed children with JIA. The trivalent vaccine had good immunogenicity and was safe to use in both JIA treatment groups.

## Introduction

Juvenile Idiopathic Arthritis (JIA) is the most common chronic rheumatic disorder in children. There are currently seven subtypes of JIA, with the most common forms being are oligoarticular (OA) and polyarticular (PA) JIA. Children with pediatric rheumatic diseases are at increased risk of infections, which can contribute to morbidity and mortality, and can also trigger a flare-up of JIA symptoms. Several factors contribute to the increased risk of infection in JIA patients, including immunological disorders associated with the disease itself and its treatment. Patients with JIA receiving immunomodulatory treatment with biological DMARDs are immunosuppressed and at increased risk of developing infections, particularly respiratory tract infections [1]. Influenza virus is a common seasonal pathogen causing acute respiratory tract infections in the pediatric population [2, 3]. Although influenza-like illnesses caused by influenza A strains are more common, illnesses caused by influenza B strains are associated with higher morbidity and mortality rates among children [4, 5]. Furthermore, young children play an important role in the community spread of influenza [6]. The Center for Disease Control and Prevention (CDC) is a major global advocate of the importance of immunization and aims to reduce the incidence of vaccine-preventable diseases (VPD). Vaccination is currently the most effective intervention to prevent infection and serious outcomes in children [7]. However, the flu virus can trigger flare-ups in autoimmune disorders, causing treatment interruption and secondary bacterial overgrowth. Therefore, the American College of Rheumatology (ACR) and the European League Against Rheumatism (EULAR) recommends that all JIA patients should receive an annual influenza vaccination [8, 9].

Based on various national guidelines, the group suggests that non-live vaccines are generally and can be administered even alongside disease-modifying antirheumatic drugs (DMARDs), corticosteroids, or biological treatments such as anti-tumor necrosis factor-alpha (anti-TNFα). The flu vaccine has been routinely introduced in only a few centers, as many doctors are not aware of the effectiveness of the vaccination when using immunosuppressive treatment and are concerned about the possibility of disease flare-ups [10]. Neverthless, vaccination with inactivated vaccines is not contraindicated during immunosuppressive treatment either.

Here, we present a clinical study investigating the immune response to influenza vaccination in children suffering with JIA receiving various medications. In addition to measuring influenza -specific antibodies peripheral blood lymphocyte subsets were also investigated. However, the primary aim of our study was not to assess the antigen-specific activation of these cells. Instead, our focus was on examining potential changes in the distribution and quantity of lymphocyte subsets following influenza vaccination. This approach is particularly important given concerns about non-specific immune activation and the potential for autoimmune disease flares following vaccination, which may discourage vaccine administration and acceptance.

## Materials and methods

We conducted a single-center prospective study from the Department of Pediatrics, Medical School, University of Pécs. Forty-one children with non-systemic JIA and 22 healthy controls were enrolled during the influenza season periods of 2019. Twenty-five (61%) children had OA and 16 (39%) had PA. Within the OA group, twelve (48%) children had persistent, and 13 (52%) extended OA. Fifteen (36.5%) among the patients (12 persistent OA, 3 PA) were treated with conventional DMARD, methotrexate (MTX) (15 mg/m2 /week orally) alone. Twenty-six (63%) (13 extended OA, 13 PA) were on a regime of MTX and anti-TNFα therapy (adalimumab [ADA], Humira, AbbVie^®^). This treatment group received MTX 15 mg/m^2^/week orally; and the dose of ADA was 20 mg (under 30 kg body weight) or 40 mg (above 30 kg body weight) administered subcutaneously every two weeks. It is important to emphasize that patients who received systemic (oral/intravenous) or intraarticular glucocorticoid therapy in the last four months prior to the start of the study were excluded. The control group consisted of healthy siblings of the treated patients. Exclusion criteria included were active arthritis, DMARD therapy other than MTX, ongoing acute illness, clinical evidence of influenza infection within the last 2 months before vaccination, and a history of previous adverse reaction or anaphylaxis to any other vaccine. Pertinent clinical data of three groups of patients are shown in Table 1.

**Table 1:** Baseline characteristics of the study population

	JIA patients treated with MTX (*n* = 15)	JIA patients treated with MTX/ADA (*n* = 26)	Healthy controls (*n* = 22)
**Age**,** mean +/- SD (years)**	7.12+/-4.81	7.47+/-4.37	12.40+/-4.02
**Males**,** no. (%)**	5 (33)	12 (46)	13 (59)
**Median duration of JIA**,** years (range)**	2.00 (0.25–13.83)	3.80 (0.91-11)	-
**Median duration of the start of therapy**,** years (range)**	2.00 (0.25–13.83)	1.88 (025-7.58)	-
**JIA subtype** - **oligo JIA**,** no. (%)** - **poly JIA**,** no. (%)**	12 (80) 3 (20)	13 (50) 13 (50)	-

The patients arrived on the day of investigation at our Clinic’s Allergy and Immunology Outpatient Care Unit. After their general clinical evaluation, a detailed rheumatologic investigation was performed and peripheral venous blood samples were taken. Serum was collected, aliquoted and stored at -80 °C. Following the sampling, all children received a whole-virion, trivalent, inactivated influenza vaccine (Fluart3^®^, Fluart Innovative Vaccines LTD, Pilisborosjenő, Hungary). The vaccine consisted of A/Michigan: 45/2015(H1N1pdm09), A/Singapore: INFIMH-16-0019-2016 (H3N2) and B/Maryland: 15/2016 (B/Victoria) types of influenza virus. The vaccine was administered intramuscularly, with age correlated dose (between 3 and 11 years 0.25 ml, over 11 years of age 0.5 ml). The vaccine was administered without interrupting therapy. None of the children had previously received a flu immunization. The follow-up visits were conducted 4 weeks and 12 weeks after vaccine administration. Between vaccination and the follow-up visits, parents documented adverse reactions and infectious diseases following vaccination in a symptom diary.

Laboratory tests were performed to measure total blood count, erythrocyte sedimentation rate (ESR), C-reactive protein (CRP), immunoglobulin (IgA, E, G, M) and complement (C3, C4, CH50-complement) levels. Peripheral blood lymphocytes were analysed by flow-cytometry. The following cell types were investigated: CD56 + natural killer cells (NK), CD3 + CD56 + natural killer T cells (NKT), CD3 + CD8 + cytotoxic- and CD3 + CD4 + helper T lymphocytes, CD3 + CD25 + activated T cells, CD3 + CD45RA + naive and CD3 + CD45RO + memory T cells, CD19 + B lymphocytes, CD19 + IgD + CD27- naive B cells, CD19 + IgD-CD27 + switched memory B cells.

The hemagglutination inhibition (HAI) assay was performed to determine the serum antibody response to influenza vaccination. All serological tests were done at a single center laboratory (Department of Virology, National Center for Public Health and Pharmacy, Budapest, Hungary).

The study was conducted according to the recommendations of the Declaration of Helsinki and the protocol was approved by the Local Ethics Committee (SN: 7387, University of Pecs, Medical School). Written informed consent was obtained from all patient’s legal guardian.

### Data analysis

Seroprotection was defined as an antibody concentration of at least 40 hemagglutination units (HAU) after vaccination. Seroconversion was defined as either a pre-vaccination HI titer < 1:10 and a post vaccination HI titer > 1:40, or a pre-vaccination HI titer ≥ 1:10 and a minimum four-fold rise in post-vaccination HI antibody titer.

The geometric mean titer (GMT) and geometric mean fold increase (GMFI) for each strains pre- and post-vaccination were also calculated.

### Statistics

Statistical analysis was performed using IBM SPSS Statistics 28 software. A descriptive statistical analysis was conducted. Continuous variables were expressed as median and interquartile range or mean ± standard deviation, depending on the distribution of the data. Normality was assessed by the Kolmogorov-Smirnov tests when appropriate. In our study, we used a mixed model to analyse data from three groups: JIA patients receiving therapy as MTX/ anti-TNFα, or MTX, and a healthy control group. All participants received the influenza vaccine and their lymphocyte subpopulations in peripheral blood were examined at three different time points. The mixed model was particularly advantageous in our research because it allowed us to account for both the fixed effects of the treatment groups and the random effects related to individual variations, providing a more accurate analysis of the immune response over time. The primary outcome of the analysis was the comparison of the distribution and quantity of lymphocyte subsets, as determined by flow cytometry, along with inflammatory laboratory parameters, across the three groups at three different time points: at vaccination, and 4- and 12- weeks post-vaccination.

The mixed model effectively handles missing data by using maximum likelihood estimation methods, allowing for accurate analysis without the need to impute missing values. Categorical data, such as the number of seroconversions and seroprotections in the three groups, were analysed using contingency tables and the chi-squared or Fisher’s exact test, as appropriate. Statistical significance was established as a p-value of < 0.05.

## Results

Altogether 41 JIA and 22 healthy children took part in the study. All JIA patients were in an inactive state of the disease at the visits.

The patient characteristics are listed in Table 1. Basic laboratory results (ESR, CRP, blood count) were within the normal range in all three groups at all three study time points (Table 2).

**Table 2:** Basic laboratory findings

	JIA patients treated with MTX	JIA patients treated with MTX/ADA	Healthy controls	Significance (*p*-value)		
				p1	p2	p3
**ESR (mm/hour)**	12.00 ± 7.82	10.85 ± 6.63	8.68 ± 5.88	0.295	0.509	0.851
**CRP (mg/l)**	0.85 ± 1.67	1.03 ± 2.13	1.09 ± 1.68	0.923	0.992	0.957
**Leukocyte (abs.)**	6920 ± 2380	7220 ± 1470	6770 ± 1350	0.959	0.630	0.846
**ANC (G/l)**	3.94 ± 1.57	3.71 ± 1.03	3.69 ± 1.14	0.803	0.999	0.814
**Monocyte (%)**	0.33 ± 0.18	0.38 ± 0.12	0.33 ± 0.84	0.988	0.429	0.392
**Thrombocyte (G/l)**	304.06 ± 62.47	295.04 ± 58.78	302.05 ± 89.23	0.996	0.939	0.917

(p1 = MTX and control, p2 = MTX/ADA and control, p3 = MTX and MTX/ADA, ANC: absolute neutrophil count)

Outcome measures were spread to humoral immune response to influenza immunisation in patients with different therapies of JIA. In the MTX/ADA group pre-vaccination seroprotection was demonstrated in 56% and 33% in 2 of 3 vaccine strains (H1N1 and H3N2 respectively). After administration of the vaccine 82% and 89% of patients showed elevated antibody levels (*p* = 0.040, *p* < 0.001) for H1N1 and H3N2 on the second visit. Post-vaccination GMT values for H1N1 and H3N2 particles indicated, effective vaccine coverage. The response to the influenza vaccine strains (type A) demonstrated protective titer of 100% for H1N1 and 80% for H3N2 after vaccination (*p* = 0.002 and *p* = 0.058) in the MTX group. GMTs were 24.40 and 78.80 (*p* < 0.001) in the case of H3N2. Data are shown in Table 3. In the control group, we found 89.47% and 84.2% protective titers for the H1N1 and H3N2 strains. In the case of serotype B influenza, seroprotection was not achieved, with an average increase in titres of 15%, 0% and 22% in the anti-TNFα therapy, MTX therapy and healthy control groups, respectively. The GMFI values for H1N1 and H3N2 provided adequate protection in all three study groups.

**Table 3:** Immunogenicity of H1N1, H3N2, and B influenza vaccine in JIA patients with or without anti-TNF therapy and healthy controls

	ADA + MTX therapy (*n* = 26)	MTX (*n* = 15) therapy	Healthy controls (*n* = 22)	*p*-values
A/Michigan (H1N1)				
Seroprotection, no (%)				
**Baseline**	15 (55.6%)	7 (46.7%)	11 (57.9%)	0.763
**Second visit**	22 (81.5%) *	15 (100%) *	17 (89.47%)*	0.155
**Third visit**	20 (74.1%)	14 (93.3%)	16 (84.2%)	0.138
**Seroconversion rate**	37%	73%	37%	0.102
**GMT**				
**Baseline**	57.60	31.43	87.37	0.243
**Second visit**	141.40 *	317.14	229.47*	0.175
**Third visit**	111.20	327.14	167.47	0.093
**A/Singapore (H3N2)**				
**Seroprotection**,** no (%)**				
**Baseline**	9 (33.3%)	7 (46.7%)	7 (36.8%)	0.708
**Second visit**	24 (88.9%) *	12 (80.0%)	16 (84.2%)*	0.205
**Third visit**	16 (59.3%)	14 (93.3%)	16 (84.2%)	0.087
**Seroconversion rate**	52%	40%	37%	0.616
**GMT**				
**Baseline**	24.40	46.43	46.31	0.705
**Second visit**	78.80 *	87.86*	80.26	0.820
**Third visit**	57.40	137.50	86.58	0.079
**B/Maryland (B/Vic)**				
**Seroprotection**,** no (%)**				
**Baseline**	1 (3.7%)	0 (0%)	1 (5.3%)	1.000
**Second visit**	4 (14.8%)	0 (0%)	4 (22.2%)	0.304
**Third visit**	4 (14.8%)	0 (0%)	2 (11.1%)	0.359
**Seroconversion rate**	7%	0%	11%	0.426
**GMT**				
**Baseline**	5.60	7.50	9.74	0.633
**Second visit**	13.20	8.57	16.84	0.382
**Third visit**	10.80	13.57	20.26	0.398

* *p* < 0.05 from baseline to second visit (within-groups)

^1^ value that provides adequate protection

When comparing the vaccine-responses among the three study groups, no significant difference was observed. Neither biologic therapy nor MTX had a negative effect on seroprotection (data available upon request). Only mild side effects such as localized pain and redness were reported, and no medical intervention was necessary. During the follow-up period, no child reported any symptoms suggestive of influenza infection.

Flow cytometry was used to compare data from lymphocyte populations in the three study groups at the three time points. As shown in Table 4, no significant difference was found when the groups and time points were analysed together. Tables 5 and 6 show the distribution (%) of lymphocytes and the absolute cell numbers analysed with flow cytometry.

Absolute lymphocyte count was found to be significantly lower in MTX-treated patients compared to the group receiving anti-TNFα. Additionally, there was a significant difference in the absolute number of CD3 + T cells between the MTX group and the group receiving biological therapy, as well as between the biological therapy group and the healthy controls. No significant difference was detected in the results for CD3/CD25+, CD3/CD45RA and CD3/CD45RO cells. Our investigation extended to other lymphocyte-cell subsets as CD19+, absolute B cell, naive and switched memory B cell and NKT cells which did not show significant difference among the groups. The absolute number of CD56+, natural killer (NK)-cells was significantly elevated in the control group compared to the group receiving anti-TNFα therapy. Alterations in lymphocyte subpopulations were analysed after vaccination. The percent value of CD3/CD25 positive cells was different comparing the first and third and second and third visit. A significant decrease in percentage values of CD3/CD45RA naïve T-cell were observed for the third time compared to for the second. Percentage of naive B lymphocyte was lower at the last examination comparing to the second. No other significant differences were found.

However, it is important to emphasize that all the above-mentioned differences were within the age-specific normal ranges for the given parameters.

**Table 4:** Interaction time*group effect

Lymphocyte subpopulation	F-value	Degree of freedom	*p*-value
**CD3+ (abs.)**	0.355	108.6	0.840
**CD3/CD25+ (%)**	0.1551	106.7	0.960
**CD3/CD45RA (%)**	0.357	106.8	0.839
**CD3/CD45RO (%)**	0.203	106.7	0.936
**CD4+ (abs.)**	0.608	107.2	0.658
**CD8+ (abs.)**	0.330	108.2	0.857
**CD19+ (abs.)**	0.0851	108.9	0.987
**Lymphocyte (abs.)**	0.254	113.4	0.907
**Naive B (%)**	1.0481	109.9	0.386
**NK cell (%)**	0.227	111.0	0.922
**NKT cell (%)**	0.894	96.0	0.471
**Switched B (%)**	0.6059	110.1	0.659

The subpopulations in all three groups at all three time points examined simultaneously using the mixed model. The interaction group effect (group × time) in our mixed model analysis examines whether the effect of time on the outcome differs between groups. The F-value quantifies how much variation in the outcome can be explained by the interaction effect (group × time) relative to unexplained variation. A larger F-value suggests a stronger interaction effect, while a small F-value suggests little to no interaction. The p-value associated with the F-test indicates whether the interaction effect is statistically significant

**Table 5:** Group factor effect

	Estimated marginal means			*p* values		
	Group 1 (MTX)	Group 2 (ADA + MTX)	Group 3 (Control)	Gr. 1 vs. 2	Gr. 1 vs. 3	Gr. 2 vs. 3
Subpopulation	Mean ± SD and 95% CI					
**CD3+ (abs.)**	1589.0 ± 832.9 1325.0-1852.0	2044.0 ± 851.3 1846.0-2242.0	1622.0 ± 834.9 1405.0-1839.0	0.023	1.000	0.017
**CD3/CD25+ (%)**	10.2 ± 5.35 8.49–11.8	10.3 ± 5.44 9.02–11.6	10.5 ± 5.31 9.08–11.8	1.000	1.000	1.000
**CD3/CD45RA (%)**	44.2 ± 17.03 38.9–49.6	43.1 ± 17.29 39.1–47.1	40.4 ± 16.9 36.0-44.8	1.000	0.824	1.000
**CD3/CD45RO (%)**	30.9 ± 14.66 26.3–35.4	32.4 ± 14.88 28.9–35.9	30.8 ± 14.59 27.0-34.6	1.000	1.000	1.000
**CD4+ (abs.)**	906.0 ± 451.57 763.0-1049.0	1119.0 ± 461.94 1012.0-1227.0	926.0 ± 452.42 809.0-1044.0	0.060	1.000	0.056
**CD8+ (abs.)**	582.0 ± 435.13 444.0-719.0	770.0 ± 445.60 667.0-874.0	582.0 ± 436.29 468.0-695.0	0.098	1.000	0.050
**CD19+ (abs.)**	210.0 ± 204.28 145.0-274.0	288.0 ± 206.46 239.0-336.0	261.0 ± 204.32 208.0-314.0	0.174	0.672	1.000
**Lymphocyte (abs.)**	2120 ± 1040 1800–2440	2700 ± 1050 2450–2940	2260 ± 1030 1990–2520	0.016	1.000	0.052
**Naive B (%)**	78.7 ± 14.82 74.2–83.3	77.8 ± 14.8 74.4–81.3	78.7 ± 14.52 74.9–82.5	1.000	1.000	1.000
**NK cell (%)**	11.9 ± 8.21 9.43–14.4	10.3 ± 8.22 8.44–12.2	14.4 ± 8.09 12.28–16.50	0.936	0.425	0.018
**NKT cell (%)**	7.02 ± 8.50 4.41–9.63	4.63 ± 8.40 2.64–6.62	5.43 ± 8.40 3.25–7.61	0.445	1.000	1.000
**Switched B (%)**	12.7 ± 9.97 9.65–15.7	12.8 ± 9.98 10.49–15.1	13.4 ± 9.83 10.84-16.0	1.000	1.000	1.000

The subpopulations in all three groups tested simultaneously using the mixed model

**Table 6:** PostHoc test between time points

	Estimated marginal means			*p* values		
	Time 1	Time 2	Time 3	Time 1 vs. 2	Time 1 vs. 3	Time 2 vs. 3
Subpopulation	Mean ± SD and 95% CI					
**CD3+ (abs.)**	1798.0 ± 463.59 1652.0-1943.0	1733.0 ± 663.24 1581.0-1886.0	1723.0 ± 583.77 1573.0-1874.0	0.960	0.727	1.000
**CD3/CD25+ (%)**	9.87 ± 2.86 8.98–10.8	9.64 ± 3.95 8.73–10.6	11.4 ± 3.52 10.48–12.3	1.000	< 0.001	< 0.001
**CD3/CD45RA (%)**	41.9 ± 9.35 39.0-44.9	44.6 ± 13.08 41.5–47.6	41.3 ± 11.60 38,2-44.3	0.090	1.000	0.021
**CD3/CD45RO (%)**	32.2 ± 7.75 29.8–34.7	30.6 ± 10.58 28.1–33.0	31.3 ± 9.45 28.8–33.7	0.067	0.571	0.953
**CD4+ (abs.)**	994.0 ± 252.35 915.0-1073.0	987.0 ± 360.44 904.0-1071.0	970.0 ± 317.99 888.0-1052.0	1.000	1.000	1.000
**CD8+ (abs.)**	667.0 ± 236.54 593.0-742.0	628.0 ± 335.49 551.0-706.0	639.0 ± 296.49 562.0-715.0	0.564	0.967	1.000
**CD19+ (abs.)**	252.0 ± 126.49 213.0-292.0	242.0 ± 185.81 199.0-284.0	264.0 ± 162.07 222.0-306.0	1.000	1.000	1.000
**Lymphocyte (abs.)**	2420 ± 600 2240–2600	2330 ± 820 2150–2520	2310 ± 720 2130–2500	0.848	0.579	1.000
**Naive B (%)**	78.9 ± 8.66 76.3–81.6	80.0 ± 11.61 77.3–82.7	76.3 ± 10.37 73.6–79.0	1.000	0.107	0.010
**NK cell (%)**	12.6 ± 4.69 11.2–14.0	12.0 ± 6.37 10.5–13.4	12.1 ± 5.69 10.6–13.5	1.000	1.000	1.000
**NKT cell (%)**	5.30 ± 5.18 3.73–6.87	5.05 ± 6.93 3.43–6.66	6.74 ± 6.27 5.14–8.35	1.000	0.237	0.125
**Switched B (%)**	11.8 ± 6.23 9.91–13.7	13.1 ± 8.41 11.16-15.0	14.0 ± 7.48 12.08–15.9	0.611	0.093	1.000

The subpopulations at all three timepoints tested simultaneously using the mixed model

## Discussion

To our knowledge, this is the first study to investigate the efficacy of vaccination and kinetics of lymphocyte subsets in patients with JIA receiving different immunomodulatory therapies. Based on our study, trivalent inactivated whole virus vaccine appears to be immunogenic, safe and effective in children with JIA, which is consistent with the results of childhood studies reported in the literature.

Assessing the vaccine response of immunocompromised individuals may provide important data to ensure safety and optimal protection. The aim of influenza vaccination is not only to achieve specific antibody titers, but also to provide protection against the influenza virus and to reduce the severity of illness if infection occurs [11]. The effectiveness of vaccines can be assessed by measuring antibody levels and calculating GMT.

The efficacy of influenza vaccination in patients receiving various immunosuppressive treatments has been studied in a relatively large number of adult patients with rheumatoid arthritis (RA). In a comparison of immune responses to influenza vaccination in RA patients treated with different DMARDs, it was observed that patients treated with MTX alone had a more robust antibody titer for influenza antigens than patients treated with TNF-alpha blockers with or without MTX. Centrum germinativum response is essential for memory B cell formation, and the anti-TNFα therapy blocked this response, thereby reducing peripheral memory B-cell numbers and consequently reducing the response to influenza vaccination detectable [12–18]. Still, patients with RA receiving anti-TNF-alpha treatment developed adequate seroprotection despite lower GMT [12]. A poor immunogenic response is observed in patients treated with steroids [14].

There is limited data on the serological response of children with JIA receiving various immunosuppressive therapies, and most studies are small case-control reports [19–24]. Our study showed a reduced antibody response to influenza B in all three study groups. This result can also be explained by a single vaccination, but it is important to emphasise that in the few studies conducted so far in JIA, two studies have also shown low influenza B titres despite 2 doses of vaccine [19, 21].

Dell’Era and colleagues used MF-59 conjugated trivalent vaccine in the JIA group, influenza B antigen GMTs, seroconversion and seroprotection rates were all significantly lower etanercept-treated group than in the MTX and healthy groups. In view of the low influenza B titres observed in some studies, antiviral medication may be needed in addition to vaccination in case of influenza B infection. Despite a lower GMT, an adequate antibody response to influenza A was observed [22].

An important observation in our study was that although the children had not previously received a flu vaccine or had no typical symptoms of influenza infection, an influenza A antibody titer was detectable at the start of the study. As in other pediatric studies, we have observed adequate GMT against influenza A [21, 22, 25]. This result suggests that neither anti-TNFα biological therapy nor MTX treatment affects the influenza A antibody response.

Changes in the distribution of lymphocyte subsets following vaccination have only been studied in adult patients so far. The naïve B-cell repertoire is crucial for the response to antigens. The non -switched B cell are generated by a T cell-independent immune response to antigens such as polysaccharides, nucleic acids and lipids, whereas class-switched B cells are generated through a T cell-dependent process that occurs primarily in lymphoid follicles. After B cells recognize an antigen and present it to helper T cells the activated T cells provide essential signals triggering class switching in B cells, allowing them to produce different antibody classes [26]. TNF influences the development of the B-cell repertoire and its responsiveness through several mechanisms [27, 28]. Studies, both with both short- and long-term follow-up of anti-TNF-treated RA patients showed reduced influenza-specific serum antibody titers compared with healthy subjects, which correlated with the reduced lower influenza-specific memory B-cell levels [18, 29]. In another study, although the influenza-specific effector B cells were significantly reduced, an adequate antibody response was seen in almost all patients [29]. No differences in total B lymphocyte counts or B lymphocyte subpopulations were observed in our patients.

DMARD treatments have different effects on T lymphocyte function. In addition to increasing the sensitivity of T cells to apoptosis, MTX inhibits NF-κB activity and suppresses Treg cells [30, 31]. Inhibition of TNF alpha binding to the surface of activated macrophages and monocytes via TNFα receptor (TNFR) reduces CD4 + T cells and thus the T cell-dependent B cell response [32]. Data on T lymphocytes have not been reported in adult studies.

Children receiving chemotherapy had significantly reduced CD3 + CD56+ (NKT-like) cell counts after vaccination [31]. This discrepancy was not confirmed in our study, which may be explained by the fact that DMARD treatment of patients with JIA is less immunosuppressive.

Based on our study results, no lymphocyte subset was identified for which routine testing is recommended after vaccination. Furthermore, our findings do not suggest non-specific immune activation following vaccination based on the distribution and quantity of the lymphocyte subsets that were investigated.

Important factor in vaccination is the effectiveness of the vaccine, as measured by the incidence of infection. In the year following vaccination, none of our patients suffered from any respiratory illness suggestive of influenza infection.

Safety is a particularly important aspect of vaccines. To date, only one case of relapse following vaccination has been reported in patients receiving biological therapy [10]. Our patients developed only local hyperemia and mild transient arthralgia after vaccination, no relapse was observed.

There are limitations in our study. It was conducted at a single center, and as a result, the number of subjects was small, which may have limited the power to detect differences between the immunocompetent and immunosuppressed groups. It was not possible to include the group receiving biological DMARD therapy without MTX. The study was not powered to investigate the efficacy of vaccination but only the immune response.

## Conclusion

The trivalent inactivated whole-virus vaccine seems to be immunogenic, safe, and effective in children with JIA. Our results support the existing experience with the use of the vaccine in pediatrics and confirm its use in patients with JIA in a single-center study.

## Data Availability

No datasets were generated or analysed during the current study.

## Abbreviations

ADA: adalimumab
anti-TNFα: anti- anti-tumor necrosis factor alfa
CD: cluster of differentiation
GMT: geometric mean titer
JIA: juvenile idiopathic arthritis
MTX: methotrexate
TIV: trivalent influenza vaccine
